# The putative forkhead transcription factor FhpA is necessary for development, aflatoxin production, and stress response in *Aspergillus flavus*

**DOI:** 10.1371/journal.pone.0315766

**Published:** 2025-03-03

**Authors:** Jessica M. Lohmar, Stephanie R. Gross, Carol H. Carter-Wientjes, Brian M. Mack, Qijian Wei, Matthew D. Lebar, Jeffrey W. Cary

**Affiliations:** Agricultural Research Service, United States Department of Agriculture, New Orleans, Louisiana, United States of America; University of California Riverside, UNITED STATES OF AMERICA

## Abstract

Forkhead transcription factors regulate several important biological processes in many eukaryotic species including fungi. Bioinformatic analysis of the *Aspergillus flavus* genome revealed four putative forkhead transcription factor genes. Genetic disruption of (*AFLA_005634*), a homolog of the *Aspergillus nidulans fhpA*/*fkhA* gene (*AN4521*), revealed that the *fhpA* gene is a negative regulator of both asexual spore production and aflatoxin B_1_ production in *A. flavus*. Furthermore, disruption of the *fhpA* gene caused a complete loss of sclerotial formation. Overexpression of the *fhpA* gene caused *A. flavus* to become more sensitive to sodium chloride whereas disruption of the *fhpA* gene did not change the ability of *A. flavus* to respond to any osmotic stress agent tested. Interestingly, both disruption and overexpression of the *fhpA* gene led to increases in sensitivity to the oxidative stress agent menadione. Overall, these results suggest that *fhpA* is an important regulator of morphological and chemical development in addition to stress response in *A. flavus*.

## Introduction

*Aspergillus flavus* is an opportunistic pathogen of humans and oil seed crops such as peanuts, corn, and cottonseed that can produce a wide variety of secondary metabolite compounds including a group of mycotoxins known as aflatoxins (AFs). Aflatoxins, particularly aflatoxin B_1_ (AFB_1_), have been shown to primarily target the liver leading to chronic diseases such as hepatocellular carcinoma [1,2]. Acute toxicity in humans due to ingestion of high levels of AFs can lead to severe illness, termed aflatoxicosis, and in some cases even death with the liver again being the major target organ [3,4]. Crops that exceed the maximum permissible levels of aflatoxins set by the regulatory agencies worldwide are commonly destroyed or significantly reduced in value leading to economic losses to farmers and exporters. Though difficult to accurately determine, estimates of potential annual economic losses to the US corn industry due to aflatoxin contamination have ranged from US$52.1 million to US$1.68 billion with greater losses depending on the extent to which predicted climate change alters environmental conditions to favor aflatoxin contamination [5]. Unfortunately, underdeveloped or developing countries often lack legislation or resources and technologies needed for stringent enforcement of standards for allowable levels of AFs in agricultural commodities, and thus bear the brunt of adverse health and economic impacts of AF contamination of food and feed commodities. It has been estimated that approximately 4.5 billion people in developing countries are at risk of chronic exposure to AFs [6]. Despite the development of AF mitigation strategies such as field application of biocontrol formulations, molecular and traditional methods of breeding crops for resistance, fungicides etc., a long-lasting solution to the presence of AFs in agriculturally important commodities has not been found leading to a need for novel strategies aimed at controlling the development, dissemination, pathogenesis, and production of AFs by *A. flavus*.

Fungi can disseminate, survive harsh environmental conditions, and infect other living organisms due to the unique developmental structures produced by each species. Asexual development in fungi found in the *Aspergillus* genus is commonly carried out through the growth of vegetative mycelium and the formation of a specific structure known as a conidiophore that contains asexual conidiospores which are easily dispersed by the wind allowing for efficient dissemination [7,8]. Additionally, *A. flavus* can make rigid structures known as sclerotia that are resistant to extreme environmental conditions such as drought or temperature extremes. Sclerotia are melanized masses of hyphae that lie dormant within the soil and germinate when conditions become favorable to produce hyphae and conidia that can grow, thrive, and re-infect a new season of crops [9,10]. *Aspergillus flavus* is also capable of producing sexual spores known as ascospores which occur when a sclerotium becomes fertilized by an *A. flavus* strain of the opposite mating type. After fertilization occurs, the diploid nucleus within the sclerotium (now termed stromata) undergoes meiosis to produce ascospores within the ascocarps that form inside of the stromata [11–13].

Secondary metabolism and development in fungi within the *Aspergillus* genus are temporally and spatially regulated by a multitude of genetic factors including transcription factors (TFs) [7,14]. Several classes of TFs exist in eukaryotic organisms, among them are a class known as the Forkhead box (Fox) family. This class of TFs contains an evolutionarily conserved winged-helix DNA binding domain that contains ~ 80–100 amino acids [15]. Due to the structural homology of the Forkhead box winged-helix domain to linker histones, some groups have suggested that this domain may bind nucleosomal DNA [16–19]. Fox TFs regulate several important biological processes in multiple eukaryotic species including fungi. For example, in yeast such as *Saccharomyces cerevisiae* and *Schizosaccharomyces pombe*, Fox TFs are critical for the normal regulation of important aspects of the cell cycle, chromosomal segregation, cytokinesis, septation, cell morphology, and repression of meiotic genes [20–25]. In filamentous fungi such as *Aspergillus nidulans*, *Cryptococcus neoformans*, *Ustilago maydis*, *Sclerotinia sclerotiorum*, *Acremonium chrysogenum*, *Penicillium chrysogenum*, *Magnaporthe oryzae* and *Beauveria bassania*, Fox TFs regulate several additional biological processes including development, secondary metabolite production, oxidative stress response, and pathogenesis [26–33].

Classes of TFs that regulate important aspects of *A. flavus*’s biology could further serve as novel genetic targets in *A. flavus* and AF mitigation strategies. Fox TFs have been shown to play an important role in regulating important biological processes in some fungal species. At the time we initiated this research there were no other reports on Fox TFs in *A. flavus*, but recently a Fox TF known as FkhC/McnB was identified and characterized prior to our work being completed [34]. For our study, we initially sought to explore the role of this important class of TFs in *A. flavus* in regulating vital aspects of *A. flavus’s* biology. We conducted a bioinformatic survey of the *A. flavus* genome to identify all putative Fox TFs. Of the four Fox TFs we identified, we chose to first functionally characterize the *fhpA* gene as the homolog of this gene was previously shown to play an important role in regulating important aspects of *A. nidulans*’s biology such as sexual development [28]. In the current study, we explored the role of *fhpA* in regulating asexual development, sclerotial production, AFB_1_ production and responses to stress agents in *A. flavus*.

## Materials and methods

### Strains utilized in study

The *Aspergillus flavus* strains utilized in this study are listed in Table 1. For propagation of conidia used as inoculum for experiments, strains were routinely grown at 30 °C with continuous white light (Philips F17T8/TL741 17W bulb) on double strength 5/2 agar (100 mL V8 juice, 40 g agar, pH 5.2 per liter of medium) unless specified differently [35]. When necessary, approximately 10 mM ammonium sulfate and 2 mg/mL uracil was supplemented into media to support growth of the *A. flavus* strains in the presence of *pyrG* and *niaD* auxotrophies. Fungal strains were maintained as 30% glycerol stocks and stored at −80°C.

**Table 1:** *Aspergillus flavus* strains used in this study.

Strain name	Genotype	Reference
AF70 Host	Δ*ku70*, Δ*pyrG*, Δ*niaD*, *ptrA* -	SRRC 1713 [36]
AF70 pPTRI (WT)	Δ*ku70*, Δ*pyrG*, Δ*niaD*, *ptrA*+	[36]
Δ*fhpA* 1	Δ*ku70*, Δ*pyrG*, Δ*niaD*, Δ*fhpA*::*ptrA*	This study
Δ*fhpA* 4	Δ*ku70*, Δ*pyrG*, Δ*niaD*, Δ*fhpA*::*ptrA*	This study
Δ*fhpA* 5	Δ*ku70*, Δ*pyrG*, Δ*niaD*, Δ*fhpA*::*ptrA*	This study
OE *fhpA* B-1	Δ*ku70*, Δ*pyrG*, Δ*niaD*, *gpdA*(*p*)^*An*^::*fhpA*::*trpC*(*t*)^*An*^	This study
OE *fhpA* 4	Δ*ku70*, Δ*pyrG*, Δ*niaD*, *gpdA*(*p*)^*An*^::*fhpA*::*trpC*(*t*)^*An*^	This study
OE *fhpA* 8	Δ*ku70*, Δ*pyrG*, Δ*niaD*, *gpdA*(*p*)^*An*^::*fhpA*::*trpC*(*t*)^*An*^	This study

SRRC #: refers to Southern Regional Research Center collection numbers.

*An*: *Aspergillus nidulans.*

### Bioinformatic analysis

The initial *Aspergillus flavus* FhpA protein sequence was obtained from FungiDB (https://fungidb.org/fungidb/app) by using the *A. nidulans* FhpA sequence as input into the fungiDB’s blast tool. The version of the NRRL3357 *A. flavus* genome used for listing gene accessions in this study can be found under the GenBank accessions GCA_000006275.3 and GCA_009017415.1. Homologous genes from the second accession, GCA_009017415.1, are listed in parentheses in Table 2. The Fox TF domain present in *AFLA_005634* was identified by inputting the *A. flavus* FhpA protein sequence into NCBI’s, Conserved Domain Database (CDD) (https://www.ncbi.nlm.nih.gov/Structure/cdd/wrpsb.cgi). Additional *A. flavus* proteins containing the FhpA Fox TF domain (Accession: pfam00250) listed in Table 2 were identified by using hmmsearch [37] with the the --cut_ga threshold option. The putative Fox TF proteins in *S. cerevisiae*, *S. pombe*, *A. nidulans*, and *A. fumigatus* were identified by using NCBI to conduct a blastP analysis with the identified *A. flavus* putative Fox TF protein sequences. The protein sequences were imported into Geneious Prime (Version 2023.0.1) in FASTA format and aligned into a multisequence alignment (MSA) using the Clustal Omega plugin, version 1.2.2 [38–40]. The Clustal Omega MSA was further used as input for generation of a ML phylogenetic tree. The ML phylogenetic tree was created utilizing the PhyML plugin version 3.3.20180621 present in Geneious [41] and was run using the LG substitution model with 1,000 bootstrap replicates.

**Table 2:** Fox TF gene accessions and common names used in phylogenetic analysis.

Species	Gene accession number	Protein accession number	Common name
*Saccharomyces cerevisiae* ^1^	YCR065W	P25364	Hcm1
	YPR104C	P39521	Fhl1
	YNL068C	P41813	Fkh2
	YIL131C	P40466	Fkh1
*Schizosaccharomyces pombe* ^2^	SPBC32H8.11	O13606	Mei4
	SPAC1142.08	O14270	Fhl1
	SPBC16G5.15c	O60129	Fkh2
	SPBC4C3.12	O43058	Sep1
*Aspergillus flavus* ^3^	AFLA_001154 (F9C07_2225972)	KAF7619529	FkhD
	AFLA_000926 (F9C07_2277854)	KAF7619298	FkhB
	AFLA_010669 (F9C07_1126508)	KAF7630038	FkhC/McnB
	AFLA_005634 (F9C07_7177)	KAF7620325	FkhA/FhpA
*Aspergillus nidulans* ^4^	AN4985	XP_662589	FkhD
	AN2854	XP_660458	FkhB
	AN8858	XP_682127	FkhC/McnB
	AN4521	XP_662125	FkhA/FhpA
*Aspergillus fumigatus* ^5^	Afu2g03050	XP_749440	FkhA/FhpA
	Afu3g10030	XP_754617	FkhD
	Afu3g11960	XP_754425	FkhB
	Afu5g05600	XP_754071	FkhC/McnB

^1^*S. cerevisiae* reference strain: 288C.

^2^*S. pombe* reference strain: 972h-.

^3^*A. flavus* reference strain: NRRL3357.

^4^*A. nidulans* reference strain: FGSC A4.

^5^*A. fumigatus* reference strain: Af293.

### Genetic modification of *A. flavus*

#### Genetic disruption of the *fhpA* gene.

Genetic disruption of the *A. flavus* Fox TF *fhpA* was carried out using a general fusion PCR based technique as previously described in Szewczyk *et al*. [42], with minor modifications (S1A Fig). All primer sequences and PCR amplicon sizes used for constructing and confirming the *fhpA* mutant strains are listed in Table 3. Briefly, the 5’ upstream and 3’ downstream fragments were amplified using PCR from wild-type (WT) *A. flavus* gDNA utilizing the primer pairs P1/P2 and P3/P4, respectively. The 2.0 kb selectable marker for the *Aspergillus oryzae* pyrithiamine resistance gene (*ptrA*) was amplified from the commercial vector pPTR1 (Takara Bio Inc., Shiga, Japan) utilizing the primer pair P5/P6. All three fragments were fused together into a single fusion PCR product using the primer pair P7/P8. Fungal transformation of the *fhpA* disruption cassettes into the *A. flavus* AF70 host transformation strain was carried out utilizing methods identical to those described in Cary *et al.* [36]. After incubation of the regeneration plates, colonies displaying resistance to pyrithiamine were subcultured and further confirmed with diagnostic PCR and qRT-PCR.

**Table 3:** Primers utilized in this study.

Primer name	Sequence (5’ to 3’)	PCR amplicon size
**Primers used to create the *A. flavus fhpA* mutant strains**		
P1: fhpA-5F	CTCCAACGCTGGCTTATTGC	1.1 kb
P2: fhpA-5R	GGGATCCCGTAATCAATTGCCCCGACGCATAGGTCCTGAGAC	
P3: fhpA-3F	CAAGAGCGGCTCATCGTCACCCCAGCGGAAGTTCAACTTGCC	1.3 kb
P4: fhpA-3R	GCCGCAGACAACAACCCTAT	
P5: pTRA-F	TGACGATGAGCCGCTCTTGC	2.0 kb
P6: pTRA-R	GGGCAATTGATTACGGG	
P7: fhpA-nestF	GTCACGCGATTCTCTTCCCA	3.6 kb
P8: fhpA-nestR	AGTGCTATAGCCATGGGGAA	
P9: Gpd-f	CTTCCGGCTCGTATGTTGTGTGG	6.8 kb
P10: Ptr-rev	ACGGGATCCCATTGGTAAC	
**Primers used to confirm *A. flavus fhpA* mutant strains**		
P1: fhpA-5F	CTCCAACGCTGGCTTATTGC	WT: 3.8 kb Δ*fhpA*: 4.4 kb
P4: fhpA-3R	GCCGCAGACAACAACCCTAT	
P11: Gpd CK1 - F	GTTGACAAGGTCGTTGCGTC	WT: None OE *fhpA*: 1.2 kb
P12: 132980 CK1 - R	CCCTTTGGAACTGCGATCCT	
**qRT-PCR primers for the *fhpA* gene expression analysis**		
B-tub qF	AGAGCAAGAACCAGACCTAC	
B-tub qR	GACGGAACATAGCAGTGAAC	
fhpA q2F	CGAAGAGAATACCACGAAAGGA	
fhpA q2R	CAAGGGAGAACAGGAAGAGAAG	
flbB F	GCGAATTTGACTGTCCACCAACATCG	
flbB R	CTACTGCTGGCAAAAGGACTAGACTG	
brlA F	CGCTTATGATGACAACGTGGA	
brlA R	GAACCATAGGAGGGCATTG	
nsdD F	GGTCATTGGAAGCTGGTCATAGGCAT	
nsdD R	GGGTATGAGCACGGTAATGGCTG	
sclR F	GGACCCCAAGGATTTCCCCG	
sclR R	GCAGCGTAGGTGGGAGACCA	
alfR F	GCGACCATCAGAGAGTCTTCCTTCA	
aflR R	GCAGAGCGTGTGGTGGTTGATTC	
aflC F	GGCTGTTGGCTGGATTTCTCAGG	
aflC R	CGCTCCATTGCCTCGTAAGTAGACAT	
aflM F	CGCTTGGCTCTCGCCTTTGAAC	
aflM R	CCATCCACCCCAATGATCTTTCCA	

#### Transformation of the AF70 host and *fhpA* mutant strain with the *pyrG* selectable marker.

To determine the competency of the AF70 host and AF70 Δ*fhpA* 1 strain as transformation hosts, transformations were carried out utilizing methods similar to those described above with minor modifications. Briefly, protoplasts of the *A. flavus* AF70 host and Δ*fhpA* 1 strains were generated as previously described in Cary *et al*. [36]. The protoplasts underwent PEG-CaCl_2_ mediated transformation with 5–6 ug of a plasmid known as pPG3J [43] that harbors the *Aspergillus parasiticus pyrG* selectable marker. The transformation mixture was plated into regeneration medium lacking uracil supplementation to select for positive transformants that contained a functional *pyrG* gene from integration of the pPG3J plasmid.

#### Overexpression of the *fhpA* gene.

To overexpress the *fhpA* gene in *A. flavus*, a pOE fhpA vector containing the *A. nidulans gpdA* promoter and *trpC* terminator, *A. flavus fhpA* coding region, and the *A. oryzae* pyrithiamine resistance gene (*ptrA*) was commercially synthesized by the company GenScript (Piscataway, NJ,USA).The primers P9 and P10 were used to amplify a 6.8 kb PCR product from the pOE fhpA vector that was further used as DNA to transform the AF70 host *A. flavus* strain. Transformation, selection, and confirmation of pyrithiamine resistant colonies was carried out in a similar manner used as those to confirm the *fhpA* disruption strains.

### Gene expression analysis

Transcript levels of the *fhpA* gene in addition to developmental and AFB_1_ biosynthetic pathway genes were determined by utilizing methods similar to those used in Lohmar *et al*., [44]. All the *A. flavus* strains from this study were inoculated into liquid PDB medium at a concentration of 1.0 x 10^6^ conidia/mL in duplicate. The cultures were incubated at 30 °C in the dark under static conditions for 3 days. Mycelia was harvested, flash frozen in liquid nitrogen, and stored at −80 °C. Fungal mycelium was lyophilized, and RNA was extracted using a RNeasy Plant Mini RNA extraction kit (Qiagen, Germantown, MD, USA). Approximately 1 µg of RNA was treated with DNAseI prior to carrying out cDNA synthesis utilizing an iScript gDNA Clear cDNA Synthesis Kit per manufacturer’s instructions (Bio-Rad, Herculues, CA, USA). Quantitative Real Time-PCR was carried out using iQ SYBR Green Supermix kit (Bio-Rad, Hercules, CA, USA) and a CFX96 Real-Time PCR detection system (Bio-Rad, Hercules, CA, USA). All primers utilized in the gene expression analysis are listed in Table 3. CT values obtained from all samples were normalized to the expression levels of the β-Tubulin gene (*AFLA_011078*) by the 2^−ΔΔCT^ method [45].

### Conidial quantifications

To determine if genetic alteration of the *fhpA* gene led to abnormal conidial production in *A. flavus,* the methods described in [44] were used with minor modifications*.* Briefly, the *A. flavus* strains were center point inoculated on potato dextrose agar (PDA) in triplicate and incubated at 30 °C under continuous white light. After 7 days of incubation, the plates were photographed, and samples were harvested for quantification of conidia. Quantification of conidia was carried out by taking two 6 mm agar pieces from each plate approximately 1.5 cm away from the center of the colony. The agar pieces were separately placed into 1 mL of 0.01% Triton-X and vortexed vigorously to release conidia into solution. Approximately, 10 µ L was loaded onto a counting slide and quantified utilizing an Olympus Automated Cell Counter Model R1 (Olympus Corporation, Shinjuku, Tokyo, Japan).

### Assessment of sclerotia production

Production of sclerotia was determined in the *A. flavus* strains by center point inoculating spore suspensions onto a sclerotium-inducing medium known as Wickerham agar (WKHM) as described in Chang *et al*., [46] (Per liter: 2.0 g yeast extract, 3.0 g peptone, 5.0 g corn steep solids, 2.0 g dextrose, 30.0 g sucrose, 2.0 g NaNO_3_, 1.0 g K_2_HPO_4_·3H_2_O, 0.5 g MgSO_4_·7H2O, 0.2 g KCl, 0.1 g FeSO_4_·7H_2_O (10-fold the original recipe), and 15.0 g agar per liter [pH 5.5]) with four replicates per strain. The cultures were incubated in continuous darkness for 7 days prior to photographing before and after washing with 70% EtOH to visualize sclerotia. Quantification of sclerotia was determined by taking three 7 mm agar pieces approximately 1 cm away from the center of the culture and physically counting the number of sclerotia present on the agar piece. Micrographs of the cultures were taken at 4X magnification to show close images of the sclerotia utilizing a SMX25 stereo microscope (Nikon, Kōnan, Tokyo, Japan).

### Analysis of AFB _1_

The methods utilized in Cary *et al*., [36] were used to assess AFB_1_ production in the *A. flavus* WT, Δ*fhpA* 1, and OE *fhpA* 4 strains with minor modifications. The fungal strains were center point inoculated on YES medium (Per 1 liter: 20 g yeast extract; 60 g sucrose, 15 g agar, pH 5.8) with 3 replicates per strain. The cultures were incubated at 30 °C in the dark for 7 days. Five agar pieces (6 mm) were excised from each YES agar culture and extracted with 1.5 ml acetonitrile: water: formic acid (80:19:1, v/v/v) on a shaker (200 rpm) for 2 h. The extracts were centrifuged (14,000 rpm) to pellet the particulate and samples were diluted 10-fold so the aflatoxin signal would not oversaturate the detector. The extracts (1 µ L injections) were analyzed on a Waters ACQUITY UPLC system with a BEH C18 1.7 μm, 2.1 mm ×  50 mm column) using fluorescence detection (Ex =  365 nm, Em =  440 nm) and a 40% methanol in water isocratic solvent system (100% MeOH was eluted between samples followed by re-equilibration at 40% methanol). Analytical standards (Sigma-Aldrich, St. Louis, MO, United States) were used to identify and quantify aflatoxin B_1_ (AFB_1_). Aflatoxin content was expressed in ppb (ng AF/g agar).

### Osmotic and oxidative stress assays

Stress response assays to determine sensitivity to various osmotic and oxidative stress reagents were carried out utilizing methods similar to those in Baidya *et al*., [47] and Lohmar *et al*., [48]. Briefly, an initial experiment testing osmotic stress response was carried using the *A. flavus* WT, Δ*fhpA* 1, and OE*fhpA* 4 strains. The strains were center point inoculated in triplicate onto 25 mL of PDA medium supplemented with 0.6 M KCl, 0.7 M NaCl, and 1 M sorbitol and incubated at 30°C under dark conditions for 6 days prior to photographing and measuring radial colony growth in centimeters (cm). To account for uneven growth on the edge of the colony, two different radial colony growth measurements were taken at two different sections of the colony and averaged together. An additional experiment was carried out in which the *A. flavus* strains were center point inoculated into 2 mL of PDA and PDA supplemented with various concentrations of NaCl (0.7 M, 1.7 M, 2.7 M, 3.7 M, and 4.7 M) in a 24-well plate in triplicate. The plates were incubated for 4 days at 30°C in the dark prior to being visualized and photographed. A final osmotic stress experiment was carried out by center-point inoculating the strains onto 10 mL of PDA and PDA supplemented with 2.7 M NaCl in 60 x 15 mm petri plates in triplicate. The cultures were incubated under the same conditions as previous osmotic stress experiments. After incubation, radial colony growth of the strains was measured in cm.

Oxidative stress response was tested by center point inoculating the same strains utilized in the osmotic stress assay onto 3 mL of PDA medium supplemented with various concentrations of menadione (0.1 mM – 1.2 mM) into 24-well plates. The cultures were incubated at 30°C under dark conditions for 3 days prior to being photographed. The experiment was carried out with three replicates per strain. A follow up experiment was carried out by center point inoculating the *A. flavus* strains onto 10 mL of PDA and PDA supplemented with 1.2 M menadione. The cultures were allowed to incubate under dark conditions for 4 days at 30°C. Radial colony growth of the cultures was measured (in cm) after incubation.

### Statistical analysis

Statistical analysis was carried out for all quantitative data using the program R version x64 4.4.2 [49]. Analysis of variance (ANOVA) in conjunction with Tukey’s post hoc test was carried out to assess statistical differences. Data was considered statistically different with a p-value ≤  0.05.

## Results

### Bioinformatic analysis of fungal Fox TFs in the *A. flavus* genome

**Fig 1:**
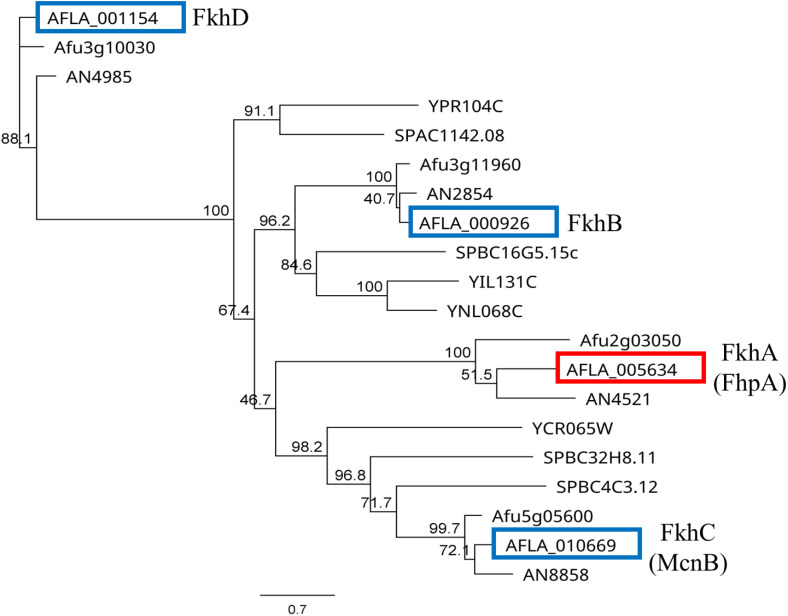
Phylogenetic analysis of fungal Fox TFs. A phylogenetic tree based on the Clustal Omega MSA was constructed using the maximum-likelihood (ML) method with a bootstrap analysis (1,000 replicates) with the support values shown at the nodes. Gene accessions listed in the tree correspond to those listed in Table 2. Boxes around the “AFLA” accession numbers correspond to the Fox TF proteins in the *A. flavus* genome.

Bioinformatic analysis of the NRRL3357 *A. flavus* genome identified *AFLA_005634* (*fkhA*/*fhpA*), *AFLA_000926* (*fkhB*), *AFLA_010669* (*fkhC*/*mncB*) *AFLA_001154* (*fkhD*) in *A. flavus* as genes that encode for putative Fox TF proteins containing the characteristic forkhead box winged helix DNA binding domain (Accession: pfam00250) (Table 2). To evaluate the evolutionary relationships between the four *A. flavus* Fox TFs and other fungal Fox TFs that have been functionally characterized, a BlastP analysis against the *Saccharomyces cerevisiae*, *Schizosaccharomyces pombe*, *Aspergillus nidulans*, and *Aspergillus fumigatus* genomes was carried out. Utilizing the identified protein sequences, a global Clustal Omega multisequence alignment (MSA) was conducted and used as input to construct a maximum-likelihood (ML) phylogenetic tree which was used to infer phylogenetic relationships (Fig 1). The resulting ML tree displayed a unique clade containing the various *Aspergillus* FhpA protein sequences which appears to have diverged from the well characterized yeast Fox TF proteins Hcm1, Mei4, and Sep1.

### *fhpA* negatively regulates the formation of asexual conidiospores in *A. flavus*

To begin to elucidate the regulatory role of the *fhpA* gene in *A. flavus*, we first disrupted the *fhpA* gene (*AFLA_005634*) in *A. flavus* in a small sclerotial (S-morphotype) strain known as AF70 [50,51]. To create AF70 Δ*fhpA* strains, we utilized a standard protoplast and CaCl_2_-PEG mediated transformation technique that selectively replaced the *A. flavus fhpA* coding region with the pyrithiamine resistance gene (*ptrA*) from *A. oryzae* by a double homologous recombination event (S1A Fig). Positive transformants were initially identified by displaying resistance to pyrithiamine and further confirmed using diagnostic PCR (S1B Fig).

Utilizing a transformation technique similar to what was used to construct the Δ*fhpA* mutant strains, we additionally attempted to generate a complementation strain by transforming a wild-type copy of the *fhpA* gene into the Δ*fhpA* 1 strain. Despite several attempts, we were unsuccessful in our efforts to create a functional *fhpA* complementation strain. Since we were unable to complement the Δ*fhpA* 1 mutant with a wild-type copy of the *fhpA* gene, we sought to determine if this mutant strain was capable of being transformed with a different fragment of DNA. Both the AF70 host strain and the Δ*fhpA* 1 mutant strain harbor a mutated *pyrG* gene creating a nutritional auxotrophy for uracil. To test the competency of the AF70 host and Δ*fhpA* 1 mutant strains, we simultaneously transformed both strains with a vector harboring a functional *pyrG* gene using the exact same procedures for both transformations. The regeneration medium plates for the AF70 host transformation yielded 68 transformants whereas the regeneration medium plates for the Δ*fhpA* 1 mutant strain yielded no transformants (S2 Fig). In addition to the inability to complement the Δ*fhpA* 1 mutant strain, these results support that this mutant strain is unable to be transformed using our standard methods for reasons unknown to us at this time.

Overexpression of a gene can cause new mutant phenotypes to arise which provide further insight on the regulatory scope of the gene being studied that cannot be provided from a traditional loss of function analysis [52]. Due to the inability to create a functional complementation strain, we instead created overexpression strains of the *fhpA* gene to study the regulatory scope of the *fhpA* gene in *A. flavus* in a more thorough manner. Overexpression of the *fhpA* gene in *A. flavus* was carried out utilizing methods similar to those used in the creation of the Δ*fhpA* strains. Briefly, a vector named pOE fhpA was constructed that placed the expression of the *fhpA* gene under the control of the constitutive *A. nidulans gpdA* promoter and *trpC* terminator (S1C Fig). OE*fhpA A. flavus* strains were created by transforming the AF70 host transformation strain with a DNA fragment containing the overexpression *fhpA* transformation cassette which was amplified from the pOE fhpA vector using PCR. Positive OE *fhpA* transformants were initially selected by resistance to pyrithiamine and diagnostic PCR (S1D Fig). In addition to diagnostic PCR, the disruption and overexpression of the *fhpA* gene in the Δ*fhpA* and OE *fhpA* mutant strains was further confirmed with qRT-PCR (S1E Fig).

**Fig 2:**
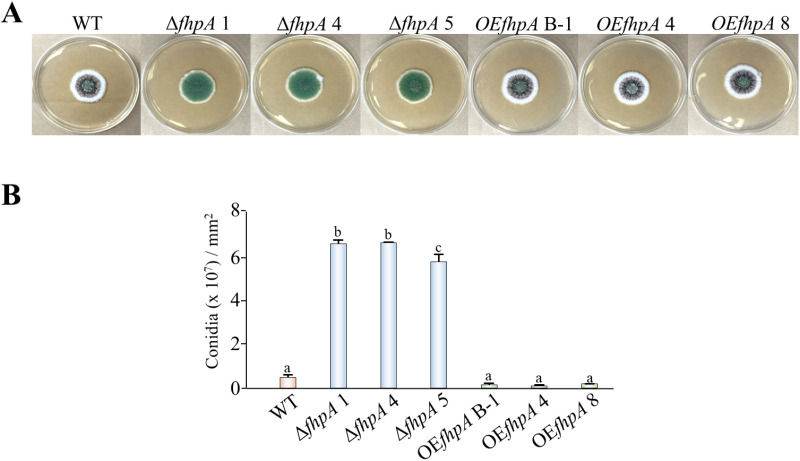
Genetic disruption of the *fhpA* gene leads to increases in conidiospore production in *A. flavus.* (A) The WT, Δ*fhpA*, and OE*fhpA A. flavus* strains were center point inoculated in triplicate onto PDA medium. Photographs of the cultures were taken after 7 days of incubation at 30 °C under light conditions. (B) Conidia was quantified in the strains by taking agar pieces from the cultures and quantifying the number of conidia present utilizing an automated cell counter. Error bars represent standard error and letters over the bars indicate statistical significance. Different letters indicate data that was statistically significant (p value ≤  0.05).

Asexual conidiospores are the most efficient form of dissemination and reproduction for fungi found within the *Aspergillus* genus and are also the primary source of inoculum during fungal infection [53]. To determine if the *fhpA* gene regulates conidiospore production in *A. flavus*, the WT, Δ*fhpA* and OE*fhpA* strains were center point inoculated onto PDA medium and grown under light conditions for 7 days prior to being photographed (Fig 2A). Quantification of conidia revealed a statistically significant increase in conidial formation in the absence of the *fhpA* gene and a decrease in conidial production when the *fhpA* gene was overexpressed that was further determined to not be statistically significant (Fig 2B).

### Sclerotial formation is positively regulated by *fhpA* in *A. flavus*

**Fig 3:**
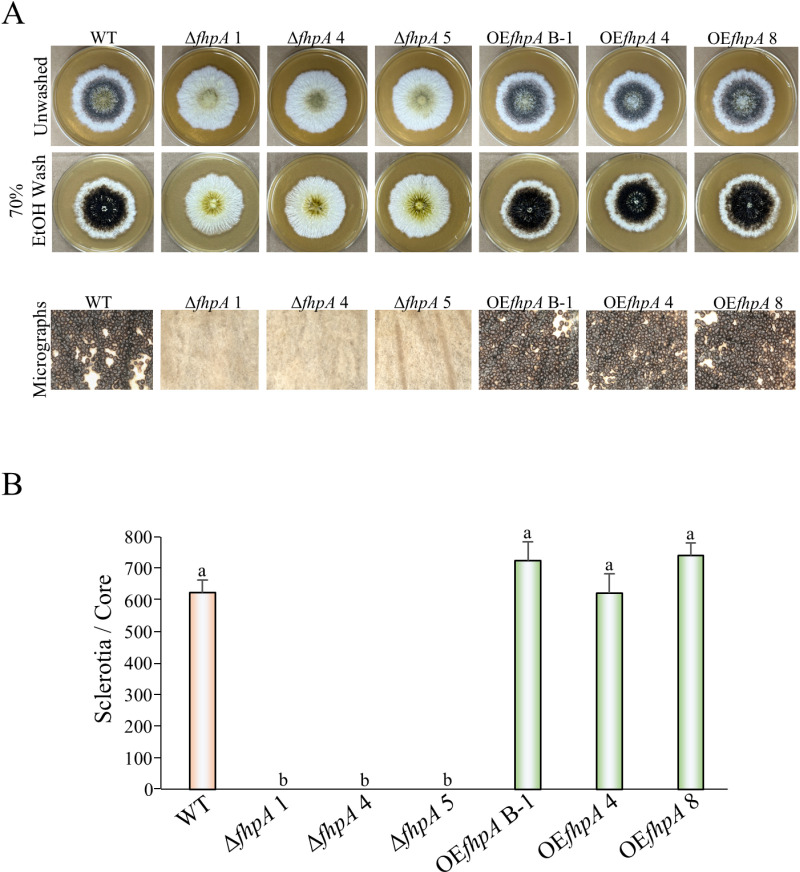
Sclerotial formation is abolished in the absence of the *fhpA* gene. (A) The *A. flavus* wild-type, Δ*fhpA* strains, and OE*fhpA* strains were center-point inoculated onto Wickerham agar medium in triplicate then incubated for 7 days at 30 °C in the dark. Photographs were taken before and after 70% ethanol washes that were used to visualize sclerotial production. Micrographs were taken of each culture after ethanol washing. (B) Quantification of sclerotia from the same cultures displayed in Fig 3, Panel A was carried out by collecting a 7 mm agar pieces approximately 1.0 cm away from the center of the colony and physically counting the number of sclerotia on each agar piece. Error bars represent standard error and letters over the bars indicate statistical significance. Different letters indicate data that was statistically significant (p value ≤  0.05).

Sclerotia are important survival structures that allow the fungus to survive harsh environmental conditions and can also become sexual developmental structures termed stromata upon fertilization by an *A. flavus* strain of the opposite mating type [9,10]. Genes encoding for putative Fox TFs have been shown to play a role in regulating sexual development in other filamentous fungi [28,29,54,55]. Due to these previous findings, we examined whether *fhpA* plays a role in regulating sclerotial biogenesis in *A. flavus*. To test this hypothesis, the same strains used to assess conidiospore production were single point inoculated WKHM medium and grown for 7 days under dark conditions. After incubation, the plates were removed from the incubator and photographed before and after washing with 70% ethanol (Fig 3A). The number of sclerotia produced by each strain was quantified by taking a 7 mm agar piece approximately 1.5 cm away from the center of the colony and physically counting the number of sclerotia present on the agar piece. Quantification of agar pieces taken from each culture revealed a complete abolishment of sclerotial production in the Δ*fhpA* strains and no statistically significant change in the amount of sclerotia produced in the OE*fhpA* strains when compared to the WT control strain (Fig 3B).

### Disruption of *fhpA* leads to increases in AFB _1_ production

Our initial assays examining the role of the *fhpA* gene in regulating asexual development and sclerotial formation in *A. flavus* used three disruption and overexpression transformants for each strain. Quantifying the amount of conidia and sclerotia in all three Δ*fhpA* transformants revealed that the transformants produced similar amounts of conidia and sclerotia when compared to the WT strain. A similar finding was seen when assessing conidial and sclerotial levels in all three OE*fhpA* transformants. Due to this, a single disruption strain and a single overexpression transformant were selected for additional experiments in our study. More specifically, the Δ*fhpA* 1 and OE*fhpA* 4 strains were selected and utilized in additional experiments.

**Fig 4:**
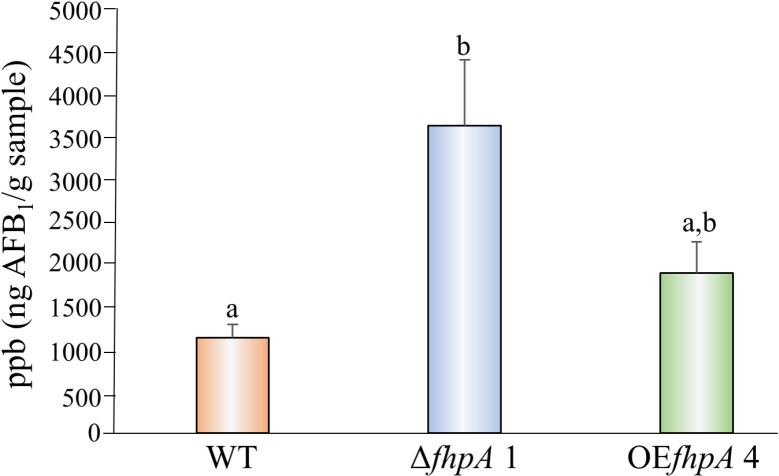
*fhpA* negatively regulates the production of AFB_1_ biosynthesis in *A. flavus.* The *A. flavus* WT, Δ*fhpA* 1, and OE*fhpA* 4 strains were center point inoculated on YES medium and incubated under dark conditions for 7 days. AFB_1_ levels were analyzed by UPLC analysis and represented as ppb (ng/g of sample). Different letters represent statistical significance (p ≤  0.05), and error bars represent standard error.

Previous reports show that Fox TFs participate in regulating the production of secondary metabolites compounds such as cephalosporin C and penicillin in *Penicillium* and *Acremonium* fungi [26,27]. *Aspergillus flavus* is well known to produce many secondary metabolite compounds including the well-known liver carcinogen AFB_1_. To determine if the *fhpA* gene regulates the production of AFB_1_, the WT, Δ*fhpA* 1, and OE*fhpA* 4 strains were single point inoculated on YES medium and incubated under dark conditions for 7 days. UPLC analysis of AFB_1_ levels in the YES cultures revealed a statistically significant increase in AFB_1_ in the Δ*fhpA* 1 strain. A slight increase in AFB_1_ was observed in the OE*fhpA* 4 strain that was not statistically different from either the WT or the Δ*fhpA* 1 strain (Fig 4).

### Overexpression of *fhpA* coincides with changes in expression of developmental and AFB _1_ regulatory genes

**Fig 5:**
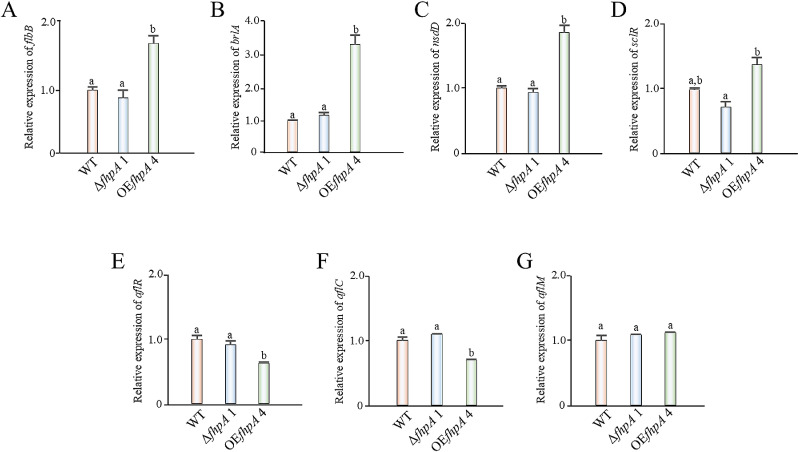
Overexpression of the *fhpA* gene affects the expression of specific developmental and AFB_1_ regulatory genes. Relative gene expression levels of (A) *flbB* (B) *brlA* (C) *nsdD* (D) *sclR* (E) *aflR* (F) *aflC* and (G) *aflM* were analyzed from mycelium that was harvested from liquid static PDB cultures that were inoculated at a concentration of 1.0 x 10^6^ spores/mL and incubated at 30°C under dark condition for 3 days. Statistical significance (p ≤  0.05) is represented by different letters placed over the top of standard error bars.

Fungal developmental processes and secondary metabolite production are temporally and spatially regulated by a multitude of genes. Due to observing developmental defects and changes in the amount of AFB_1_ produced by the Δ*fhpA* 1 strain, we wanted to next determine if the expression of specific developmental and AFB_1_ regulatory genes were altered in the absence or overexpression of the *fhpA* gene in *A. flavus*. Due to the vast number of known developmental and AFB_1_ regulatory genes, we chose to only assess the expression of *flbB*, *brlA*, *nsdD*, *sclR*, *alfR*, *alfC,* and *aflM* in the WT, Δ*fhpA* 1, and OE *fhpA* 4 strains. Asexual development is a process that is regulated by a multitude of genetic factors including the bZIP TF *flbB* and C_2_H_2_ TF *brlA* which are part of the *A. nidulans* upstream developmental pathway (UDP) and central developmental pathway (CDP), respectively [56]. The GATA TF *nsdD* and HLH TF *sclR* are known to regulate sclerotial formation in *A. flavus* and *A. oryzae* [57,58]. Additionally, *alfR*, *aflC,* and *aflM* are genes within the AFB_1_ biosynthetic gene cluster and have been shown to be indispensable for normal AFB_1_ biosynthesis. More specifically, *aflR* is the AF pathway trasncription factor that co-regulates the expression of the entire biosynthetic gene cluster with another TF known as *aflS*(*aflJ*), *alfC* encodes for a polyketide synthase (PKS) gene that synthesizes the polyketide backbone of AFB_1_, and *aflM* encodes for a dehydrogenase enzyme that is necessary for the conversion of versicolorin A into dimethylsterigmatocystin at the distal end of the AF biosynthetic gene cluster [59]. The results of the gene expression analysis revealed no significant change in expression levels for any of the genes assayed in the Δ*fhpA* 1 strain when compared to the WT strain. In contrast, overexpression of the *fhpA* gene led to statistically significant changes in gene expression for all genes assayed except for *aflM* (Fig 5A–5E).

### Normal responses to osmotic and oxidative stress agents are modulated by *fhpA*

**Fig 6:**
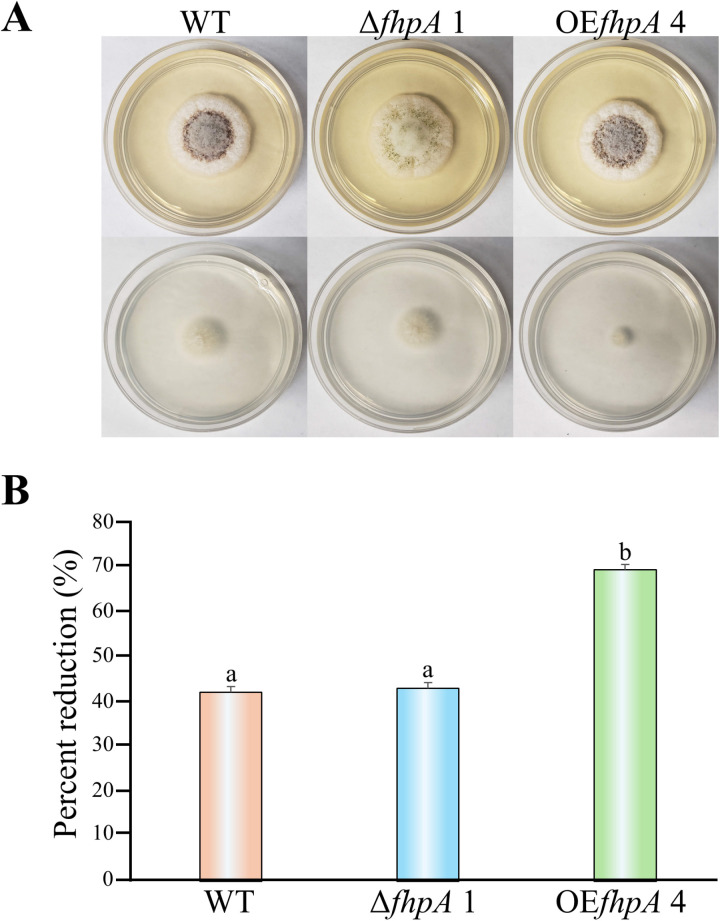
Overexpression of the *fhpA* gene alters the ability to *A. flavus* to tolerate high concentrations of sodium chloride. (A) The *A. flavus* strains were center point inoculated onto PDA medium with and without 2.7 M NaCl in triplicate. The cultures were grown for 4 days under dark conditions at 30°C prior to being photographed. (B) Radial colony growth of the cultures was measured (in cm). Data are represented as the percentage of reduction in the growth of strains on PDA medium versus PDA medium containing 2.7 M NaCl. Error bars represent the standard error. Columns with different letters represent values that are statistically different (P <  0.050).

Fungi encounter a wide variety of biotic and abiotic stresses in the environment, and they must be able to quickly adapt to various stressors to survive. Fox TFs have been reported to regulate a wide variety of stress responses in other fungi such as osmotic stress, oxidative stress response, heat stress, and conidial stress tolerance [30,33,55,60]. Due to this, we sought to determine if *fhpA* regulated osmotic or oxidative stress response in *A. flavus*. To test for abnormal responses to osmotic stress agents, the WT, Δ*fhpA* 1 and OE*fhpA* 4 strains were inoculated onto PDA supplemented with 0.6 M KCl, 0.7 M NaCl, and 1 M sorbitol and grown for 6 days under dark conditions prior to being photographed (S3A Fig). No difference in radial colony growth was observed in any culture except for the OE*fhpA* 4 strain which displayed a statistically significant reduction in radial colony growth when compared to the WT strain when exposed to 0.7 M NaCl indicating an increase in sensitivity (S3B Fig). To assess the NaCl sensitivity of the OE*fhpA* 4 strain further, the *A. flavus* strains were center point inoculated onto 2 mL of PDA and PDA supplemented with various concentrations of NaCl and incubated under dark conditions for 3 days before visually inspecting the plate for reductions in growth and photographing the plates (S3C Fig). Visual inspection of the plate showed a larger reduction in colony growth in the OE*fhpA* 4 mutant when compared to the WT strain when exposed to 2.7 M NaCl further confirming that the OE*fhpA* 4 strain is indeed sensitive to NaCl (S3C Fig). No growth of any strain was observed at NaCl concentrations of 3.7 M and 4.7 M. To determine if the sensitivity to 2.7 M NaCl observed in the OE*fhpA* 4 strain was significant, a final experiment was carried out consisting of inoculating the *A. flavus* strains onto 10 mL of PDA and PDA medium supplemented with 2.7 M NaCl only. After incubation, the cultures were photographed, and radial colony growth was measured (Fig 6A). The increase in sensitivity to 2.7 M NaCl observed in the OE*fhpA* 4 strain was determined to be statistically significant as measured by percent reduction of growth compared to the growth of each strain on PDA medium not supplemented with 2.7 M NaCl (Fig 6B)

**Fig 7:**
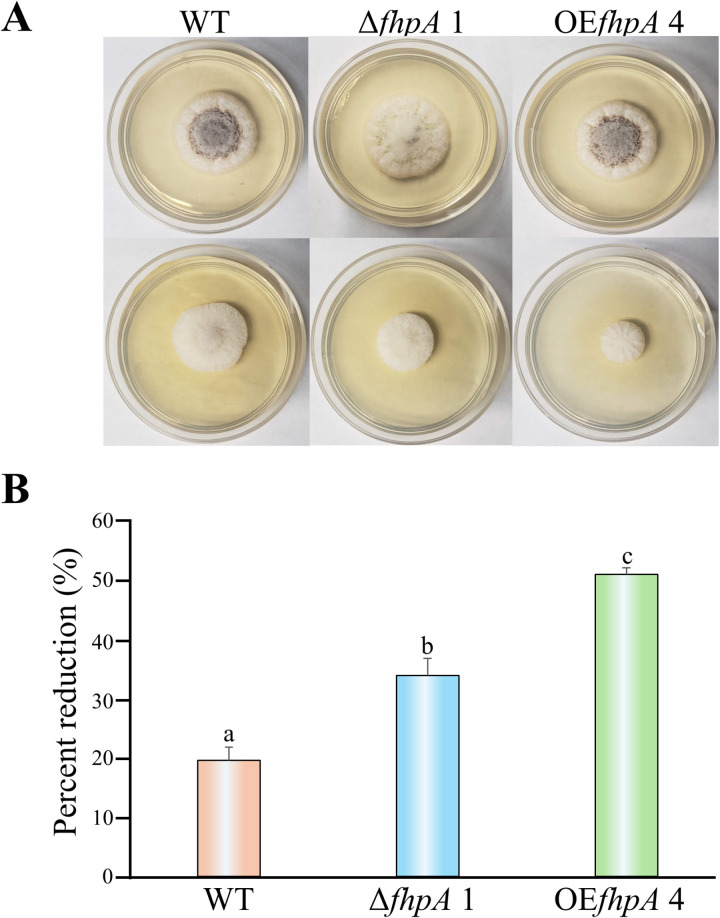
Disruption and overexpression of the *fhpA* gene increases *A. flavus*’s sensitivity to oxidative stress. (A) The *A. flavus* WT, Δ*fhpA* 1, and OE*fhpA* 4 strains were center point inoculated in triplicate onto PDA medium with and without 1.2 M menadione supplementation. The cultures were incubated in the dark for 4 days at 30°C prior to being photographed. (B) After incubation, the radial colony growth of each culture was measured (in cm). Data are represented as the percentage of reduction in the growth of strains on PDA medium versus PDA medium containing 2.7 M NaCl. Error bars represent the standard error. Columns with different letters represent values that are statistically different (P <  0.050).

To determine if genetic alteration of the *fhpA* gene altered the ability of *A. flavus* to response normally to oxidative stress, the WT, Δ*fhpA* 1 and OE*fhpA* 4 strains were center point inoculated onto PDA and PDA supplemented with various concentrations of menadione in a 24-well plate. The cultures were incubated for 3 days under dark conditions prior to being photographed. Visual inspection of the plates revealed an observable reduction in vegetative colony growth in the OE*fhpA* 4 strain when compared to the WT starting at 0.4 mM of menadione and increasing as concentration of menadione increased to 1.2 mM (S4 Fig). An additional experiment was carried out consisting of growing the *A. flavus* strains on PDA and PDA supplemented with 1.2 M menadione for 4 days under dark conditions at 30°C. After incubation, the plates were photographed, and measurements of radial colony growth were taken (Fig 7A). Interestingly, both the Δ*fhpA* 1 and OE*fhpA* 4 strains displayed statistically significant increases in sensitivity to 1.2 M menadione as measured by percent reduction of growth compared to the growth of each strain on PDA medium not supplemented with 1.2 M menadione (Fig 7B)

## Discussion

Proteins belonging to several different classes of transcription factors (TFs) have been functionally characterized in *Aspergillus flavus* and found to regulate important biological processes such as development, secondary metabolism, stress response and pathogenesis. Examples of different classes of TFs and their encoded proteins are Homeobox TFs (Hbx1), Cys_2_His_2_ zinc finger TFs (MtfA, NsdC), GATA zinc finger TFs (NsdD), and Zn_2_Cys_6_ zinc finger TFs (AswA) [36,57,61,62]. Like other classes of TFs, Fox TFs have been found to regulate several important biological processes in different yeast and filamentous fungal species [20,22,24,28–33]. It is widely known that genetic factors such as TFs undergo significant evolutionary rewiring in different organisms leading to changes in the genetic regulatory output and subsequent regulation of various biological processes leading to phenotypic alterations. Functionally characterizing specific genes on a species-by-species basis is necessary to determine if the genetic regulation of a specific gene is conserved between different fungal species or if it has been evolutionarily rewired leading to an alteration in genetic regulation and a different regulatory or phenotypic output.

In this study, we sought to determine what putative Fox TFs exist in the *A. flavus* genome and what their regulatory role is regarding processes that are important for dissemination and survival of *A. flavus* in the environment. Our bioinformatics analysis revealed the existence of the putative Fox TFs *AFLA_005634* (*fkhA*/*fhpA*), *AFLA_000926* (*fkhB*), *AFLA_010669* (*fkhC*/*mncB*) *AFLA_001154* (*fkhD*). In *A. flavus*, genetic disruption of the *fhpA* gene leads to increases in conidiation and AFB_1_ production in addition to a complete abolishment of sclerotial formation. In comparison to other *fhpA* orthologs that have been functionally characterized to date, the negative regulatory control that the *fhpA* gene has over conidiogenesis and AFB_1_ production in *A. flavus* is unique. As mentioned previously, sclerotia can become sexual developmental structures termed stromata. Stromata are known to be vestigial structures of sexual reproductive bodies called cleistothecia produced by other *Aspergillus* species, including the model fungus *A. nidulans* [63,64]. Lee and collaborators [28] reported that loss of the *fhpA* gene allowed for an abundant number of cleistothecial nursing cells known as hülle cells to form, but caused a complete abolishment of cleistothecial production in *A. nidulans*. An additional study by Fan and collaborators [29], showed that the *Sclerotinia sclerotiorum SSFoxE2* gene is an ortholog of the *fhpA* gene in *A. nidulans*. Unlike aspergilli*, S. sclerotiorum* is known to produce a different type of sexual fruiting body known as an apothecium. When *SSFoxE2* was genetically disrupted in *S. sclerotiorum,* a loss of apothecial production was observed [54]. Collectively, these results imply that *fhpA* not only has conserved regulatory control over the production of sexual developmental structures in aspergilli, but it is also conserved with *fhpA* orthologs found in more phylogenetically distant species such as *S. sclerotiorum*.

The eukaryotic cell cycle is a complex regulatory system that is regulated by a multitude of processes including negative and positive feedback loops that help regulate cell cycle check points, generate cell cycle oscillations, and make cell cycle transitions move in a single direction [65]. Normal cell cycle progression is dependent on proper spatiotemporal regulation of specific regulatory proteins. Additionally, feedback loops are common regulatory elements in almost all biological signaling systems that function to help maintain proper spatiotemporal regulation of important proteins including those involved in regulating the cell cycle. Without regulatory feedback loops to keep the cell cycle functioning as it normally does, aberrant cell cycle regulation and progression can occur leading to uncontrolled cell division in addition to abnormal changes to critical developmental programs in the cell [66]. Fox TFs have been reported to regulate important aspects of the cell cycle in yeast by regulating clusters of genes responsible for cell division and by controlling exact transcription timing of replication origins [67–72]. In higher eukaryotes, Fox TFs have also been shown to be regulated by various negative and positive feedback loops [73–76]. In fungi it is possible that the expression of specific Fox TF genes is regulated by a series of positive and negative feedback loops that are necessary to maintain proper spatiotemporal regulation of the cell cycle. In contrast to genetic disruption of the *fhpA* gene, over expression of the *fhpA* gene did not induce any phenotypes related to conidogenesis, sclerotial formation or AFB_1_ production in *A. flavus*. It is likely that overexpression of the *fhpA* gene yielded no phenotypic alterations simply due to the expression of the *fhpA* gene being regulated through a yet uncharacterized negative feedback loop which prevented the expression of the *fhpA* gene from becoming high enough to lead to noticeable alterations in morphological development and AFB_1_ production.

In addition to visually observing the Δ*fhpA* and OE*fhpA* strains for mutant phenotypes, a gene expression analysis was conducted to assess expression levels of known developmental and AFB_1_ regulatory genes to determine if any alterations in gene expression occurred due to the absence or overexpression of the *fhpA* gene. Overall, the gene expression analysis resulted in gene expression trends that did not match the developmental or AFB_1_ phenotypes observed in the *fhpA* mutant strains. In addition to this study, to our knowledge *fhpA* and orthologs of *fhpA* (*Fox1*/*SSFoxE2*) have only been characterized in *A. nidulans*, *M. oryzae*, *S. sclerotiorum*, and *U. maydis* [28,30,31,54]. None of these studies have conducted any gene expression analyses of fungal developmental or secondary metabolite genes so it is currently not known how the loss or overexpression of the *fhpA* gene directly affects the expression of specific regulatory genes in filamentous fungi.

Gene expression profiles not matching observed mutant phenotypes is not an uncommon phenomenon. An example of this phenomenon is observed when a gene known as *rtfA* is disrupted in *A. flavus* [44]. In *S. cerevisiae* the RtfA ortholog, Rtf1, has been shown to encode for a putative RNA Pol II transcription elongation factor that is known to be involved in epigenetic histone modifications. Lohmar and collaborators [44] previously conducted a time course gene expression analysis using the *A. flavus rtfA* disruption strain and isogenic control strains that revealed expression profiles of known developmental and AFB_1_ regulatory genes that did not match the mutant phenotypes observed in the Δ*rtfA* strain [44]. As mentioned previously, biological processes are spatially and temporally regulated in the cell [7]. Epigenetic regulatory proteins such as RtfA are known to contribute setting those spatiotemporal parameters in *A. flavus* and disruption one of these proteins may cause gene expression profiles to not match mutant phenotypes [44]. Previous studies have shown that the Forkhead box winged-helix DNA binding domain present in Fox TFs has structural homology to linker histones suggesting that this domain may bind nucleosomal DNA [16–19]. Recently, the Fox TF protein FoxA1 was shown to bind condensed chromatin and initiate the opening of local chromatin for gene expression in human cell lines indicating Fox TF proteins are involved in epigenetic chromatin modifications [77]. In *A. flavus*, it is possible that FhpA regulates asexual development, sclerotial formation, and AFB_1_ production through a yet uncharacterized epigenetic mechanism which alters nucleosomal proteins leading to changes in the timing of gene expression that would have not been detected in our gene expression analysis.

Additionally, due to the vast number of known developmental and secondary metabolic regulatory genes that regulate specific fungal biological process over a period of time, our study only assayed the expression of a few genes at a single time point using qRT-PCR. It is also possible that FphA modulates asexual development, sclerotial formation, and AFB_1_ production in *A. flavus* through the regulation of genes not assayed in our study. Future studies will be focused on conducting a comparative transcriptomic analysis to assay as many known regulatory genes as possible to identify known regulatory genes whose expression may be controlled by FhpA in *A. flavus*.

For fungi to survive and thrive in the environment, they must be capable of continually adjusting their physiology to quickly adapt to changes encountered in nature that could either be caused by natural causes or man-made. Fungi can adapt to rapidly changing environments and external stimuli such as temperature, pH, nutrient availability, oxidative stress, and osmotic stress due to complex signal transduction pathways that allow the fungus to respond appropriately to changes encountered in the environment [78]. Fungal Fox TFs have been reported to be involved in regulating stress response in fungi [30,33,55,60]. Due to this, we sought to determine if the *fhpA* gene regulates stress response in *A. flavus*. We carried out our analysis by exposing the *fhpA* mutants various osmotic stress agents and the oxidative stress agent menadione to look for sensitivity or resistance as indicated by differences in radial colony growth. Our results demonstrate no change in osmotic stress response in the absence of the *fhpA* gene and an increase in sensitivity to NaCl when the *fhpA* gene is over expressed demonstrating that *fhpA* is a regulator of osmotic stress response in *A. flavus*. It is likely the Δ*fhpA* strain did not display changes in sensitivity to the tested osmotic stress agents due to another unknown gene or signaling cascade having a redundant function that compensated for the absence of the *fhpA* gene. Interestingly, both genetic disruption and overexpression of the *fhpA* gene led to increases in sensitivity to the oxidative stress agent menadione indicating that *fhpA* plays a role in regulating oxidative stress response in *A. flavus*.

In conclusion, our bioinformatic analysis identified four genes in the *A. flavus* genome that are predicted to encode for putative Fox TF proteins. Through disruption and overexpression studies of the *fhpA* gene in *A. flavus*, our results demonstrate that the *fhpA* gene is an important regulator of asexual development, sclerotial formation, AFB_1_ biosynthesis and stress response in this agriculturally relevant species. This study of Fox TFs in *A. flavus* contributes to the knowledge of Fox TFs and their regulation of processes that are critical to the dissemination and survival of *A. flavus* in the environment. Future studies will be focused on functionally characterizing the remaining Fox TFs *fkhB* and *fkhD* to determine if they also play important roles in regulating *A. flavus*’s biology.

## Supporting information

S1 Fig Construction and confirmation of the *fhpA* mutant strains. (A) General construction and confirmation schematic used to disrupt the *fhpA* gene in *A. flavus*. All primer sequences and PCR amplicon sizes for P1 - P8 are listed in Table 3. Abbreviations: US – upstream sequence; DS: downstream; CDS – coding sequence. (B) Diagnostic PCR confirmation of the *A. flavus* Δ*fhpA* strains. Primer sequences and expected PCR amplicon sizes for WT and *fhpA* mutants are listed in Table 3. Location of primer binding sites is listed in panel A. M: DNA Marker (New England Biolabs, Catalog number: N3272S). (C) General construction schematic of the OE*fhpA* strains. (D) Diagnostic PCR image confirmation of the OE*fhpA* strains. Primer sequences are listed in Table 3 and binding sites in addition to expected amplicon size are shown in Panel C. M: DNA Marker (Thermo Scientific, Catalog number: SM1553). (E) Relative expression levels of *fhpA* present in the WT, Δ*fhpA*, and OE*fhpA* strains after the cultures were grown under static conditions in liquid PDB medium at 30 °C for 3 days in the dark. Error bars represent standard error. Different letters above the bars indicate statistical significance (p ≤  0.05).
(TIF)

S2 Fig The Δ *fhpA* 1 strain is unable to be transformed due to unknown reasons. The AF70 host transformation strain that was used to derive the Δ*fhpA* and OE*fhpA* strains was transformed with a vector known as pPG3J harboring the *Aspergillus parasiticus pyrG* selectable marker gene using our standard protoplast and CaCl_2_-PEG mediated transformation protocol. An identical transformation was simultaneously carried out using the Δ*fhpA* 1 strain as the host transformation strain. After transformation and incubation of the regeneration plates, colony numbers presented in the table was assessed by physically counting the number of colonies present on the plates.
(TIF)

S3 Fig Bioassays testing the susceptibility to osmotic stress agents. (A) The *A. flavus* strains were center point inoculated on PDA and PDA medium supplemented with various osmotic stress agents prior being incubated under dark conditions at 30 °C for 6 days. (B) Radial colony growth measurements were taken from the cultures displayed in panel A by measuring the diameter of the colony (in cm) at two separate areas of the colony to account for uneven edges of the colony. Statistical significance (p ≤  0.05) is represented by different letters placed over the top of standard error bars. (C) An additional experiment was performed that consisted of center point inoculating the same strains used in panel A onto PDA and PDA supplemented with various concentrations of just NaCl in a 24-well plate in triplicate. The plates were incubated under dark conditions for 3 days prior to being observed for visual reductions in growth and photographed.
(TIF)

S4 Fig Oxidative stress tolerance test. The *A. flavus* WT, Δ*fhpA* 1, and OE*fhpA* 4 strains were center point inoculated in a 24-well plate containing PDA medium and PDA medium supplemented with various concentrations of menadione in triplicate. The plates were incubated under dark conditions at 30°C for 3 days prior to being observed for reductions in vegetative growth and photographed.
(TIF)

S1 Raw Images File The S1_raw images Adobe Acrobat PDF file is a file that contains the raw unedited diagnostic PCR images utilized to make S1 Fig . (PDF)

S1 Raw Data Calculations File The raw data_calculations Microsoft Excel file contains all the raw numerical data and calculations that were used to make figure 2, figure 3, figure 4, figure 5, figure 6, figure 7, S1 Fig , and S3 Fig . Additionally, all P-values used to determine statistical significance have also been included in this file.
(XLSX)
