# Supplementary figures and images for: The putative forkhead transcription factor FhpA is necessary for development, aflatoxin production, and stress response in Aspergillus flavus
Source: PLoS One. 2025 Mar 3;20(3):e0315766. doi: 10.1371/journal.pone.0315766 (PMC11875336; doi:10.1371/journal.pone.0315766)

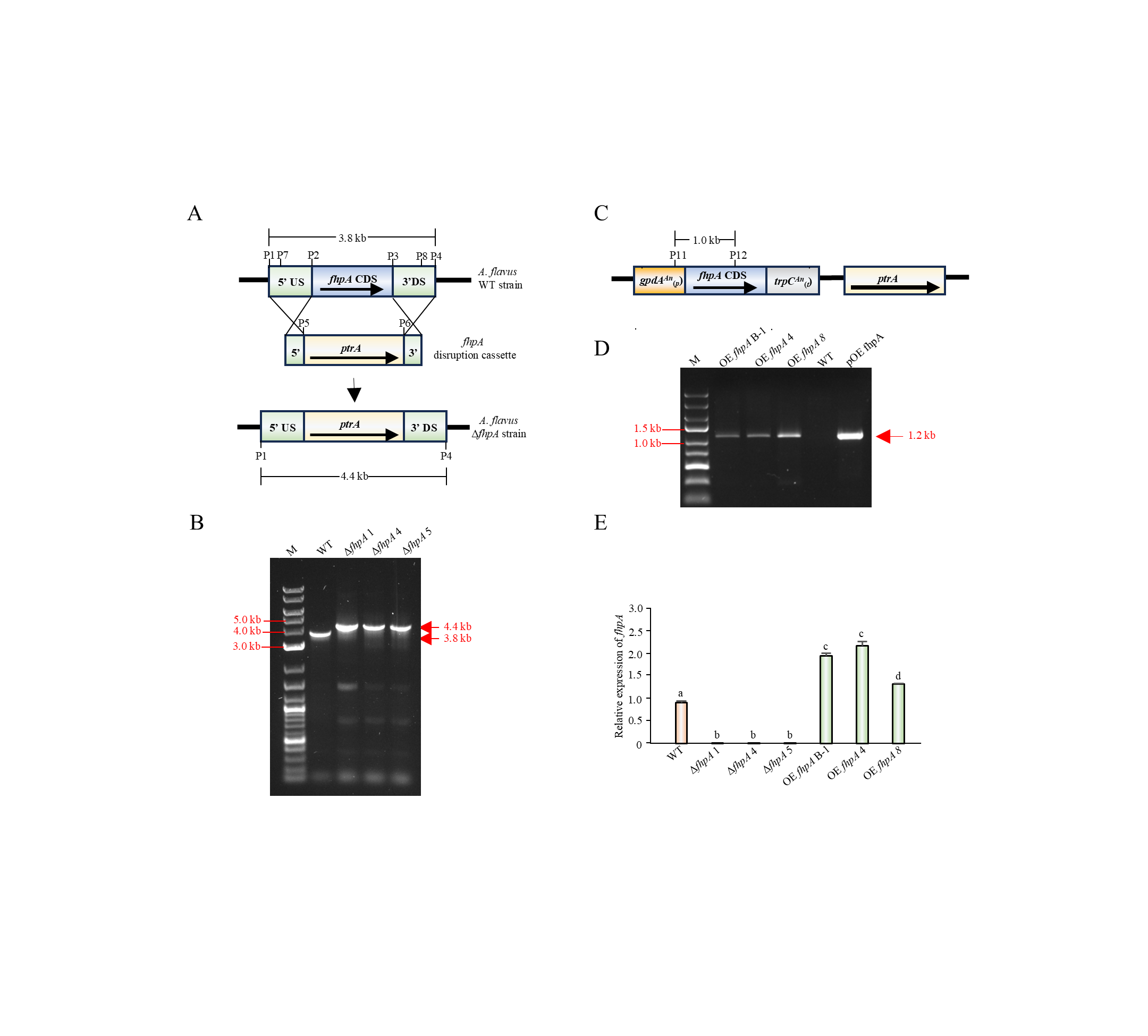

Supplement: S1 Fig — (A) General construction and confirmation schematic used to disrupt the fhpA gene in A. flavus. All primer sequences and PCR amplicon sizes for P1 - P8 are listed in Table 3. Abbreviations: US – upstream sequence; DS: downstream; CDS – coding sequence. (B) Diagnostic PCR confirmation of the A. flavus ΔfhpA strains. Primer sequences and expected PCR amplicon sizes for WT and fhpA mutants are listed in Table 3. Location of primer binding sites is listed in panel A. M: DNA Marker (New England Biolabs, Catalog number: N3272S). (C) General construction schematic of the OEfhpA strains. (D) Diagnostic PCR image confirmation of the OEfhpA strains. Primer sequences are listed in Table 3 and binding sites in addition to expected amplicon size are shown in Panel C. M: DNA Marker (Thermo Scientific, Catalog number: SM1553). (E) Relative expression levels of fhpA present in the WT, ΔfhpA, and OEfhpA strains after the cultures were grown under static conditions in liquid PDB medium at 30 °C for 3 days in the dark. Error bars represent standard error. Different letters above the bars indicate statistical significance (p ≤ 0.05). (TIF) [file pone.0315766.s001.tif]

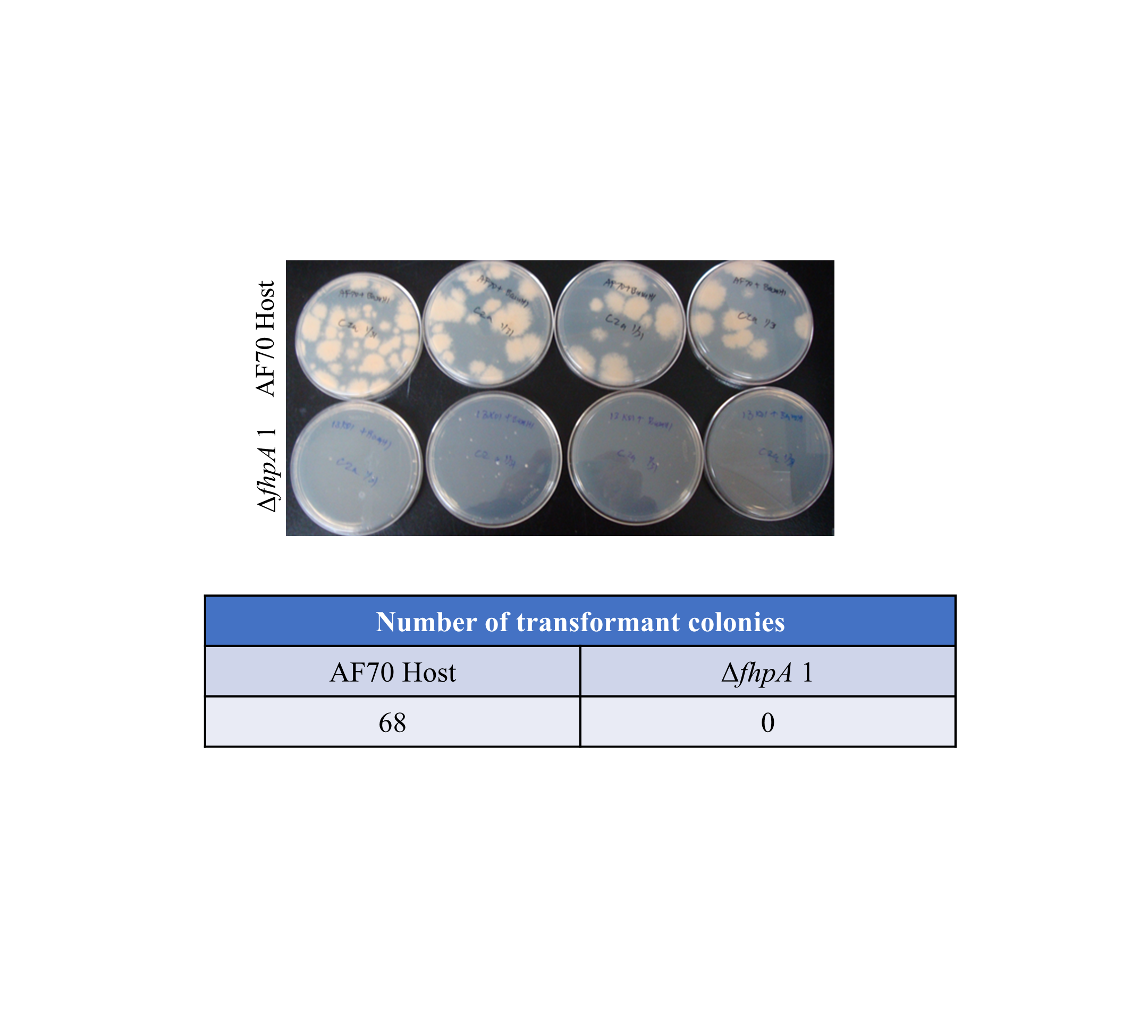

Supplement: S2 Fig — The AF70 host transformation strain that was used to derive the ΔfhpA and OEfhpA strains was transformed with a vector known as pPG3J harboring the Aspergillus parasiticus pyrG selectable marker gene using our standard protoplast and CaCl2-PEG mediated transformation protocol. An identical transformation was simultaneously carried out using the ΔfhpA 1 strain as the host transformation strain. After transformation and incubation of the regeneration plates, colony numbers presented in the table was assessed by physically counting the number of colonies present on the plates. (TIF) [file pone.0315766.s002.tif]

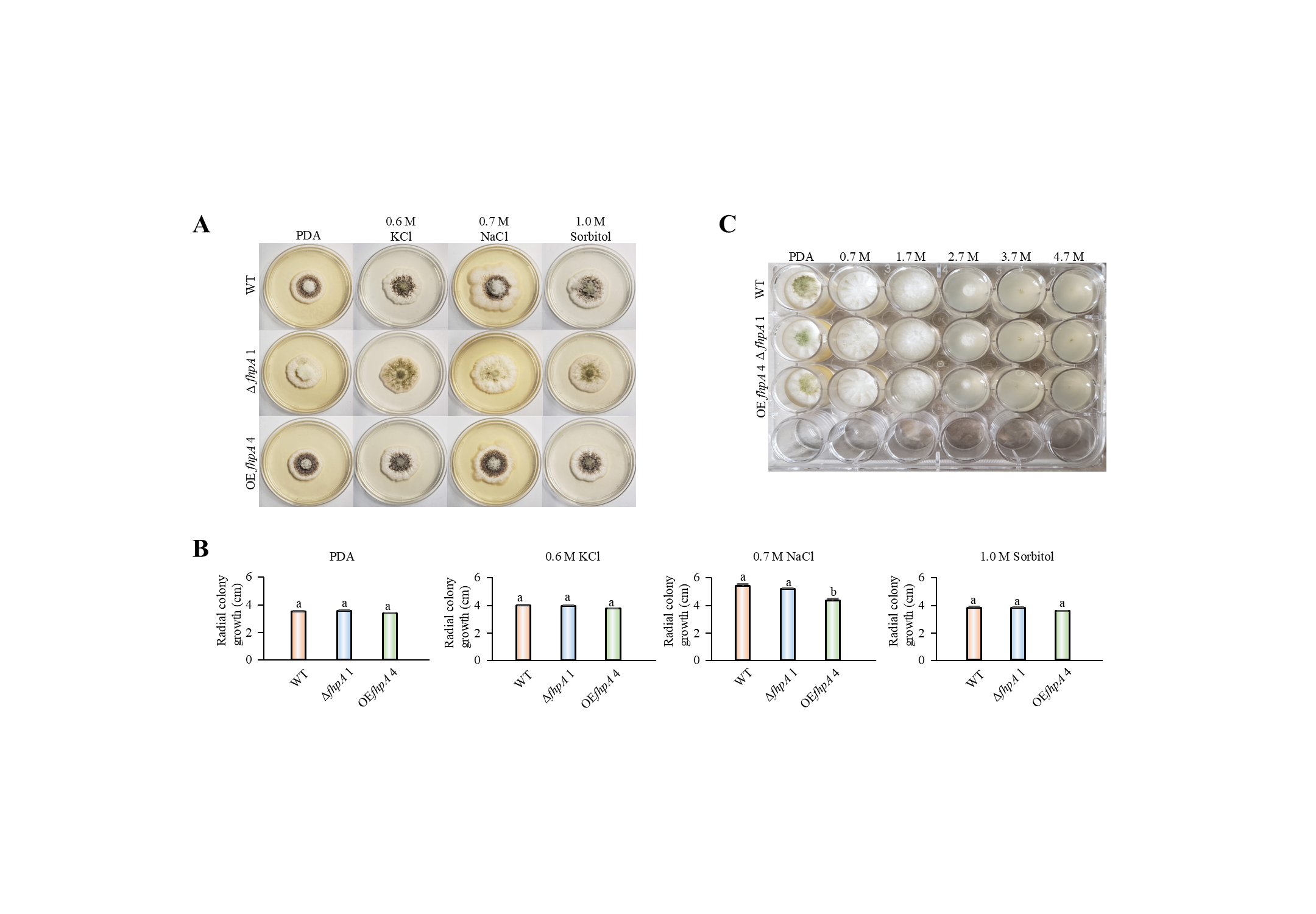

Supplement: S3 Fig — (A) The A. flavus strains were center point inoculated on PDA and PDA medium supplemented with various osmotic stress agents prior being incubated under dark conditions at 30 °C for 6 days. (B) Radial colony growth measurements were taken from the cultures displayed in panel A by measuring the diameter of the colony (in cm) at two separate areas of the colony to account for uneven edges of the colony. Statistical significance (p ≤ 0.05) is represented by different letters placed over the top of standard error bars. (C) An additional experiment was performed that consisted of center point inoculating the same strains used in panel A onto PDA and PDA supplemented with various concentrations of just NaCl in a 24-well plate in triplicate. The plates were incubated under dark conditions for 3 days prior to being observed for visual reductions in growth and photographed. (TIF) [file pone.0315766.s003.tif]

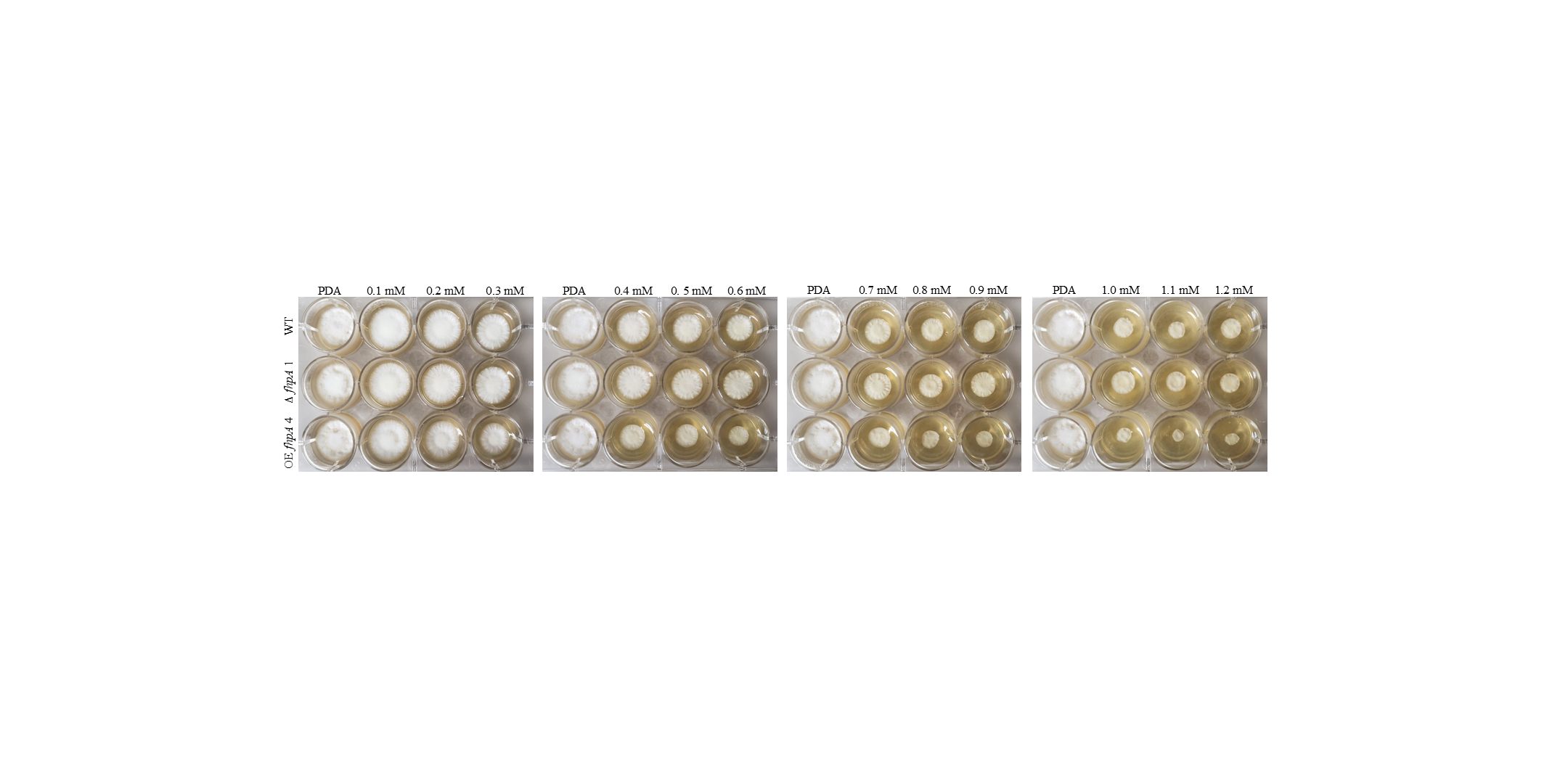

Supplement: S4 Fig — The A. flavus WT, ΔfhpA 1, and OEfhpA 4 strains were center point inoculated in a 24-well plate containing PDA medium and PDA medium supplemented with various concentrations of menadione in triplicate. The plates were incubated under dark conditions at 30°C for 3 days prior to being observed for reductions in vegetative growth and photographed. (TIF) [file pone.0315766.s004.tif]
