# Supplementary material for: The putative forkhead transcription factor FhpA is necessary for development, aflatoxin production, and stress response in Aspergillus flavus
Source: PLoS One. 2025 Mar 3;20(3):e0315766. doi: 10.1371/journal.pone.0315766 (PMC11875336; doi:10.1371/journal.pone.0315766)
Supplement: S1 Raw Images File — (PDF) [file pone.0315766.s005.pdf]

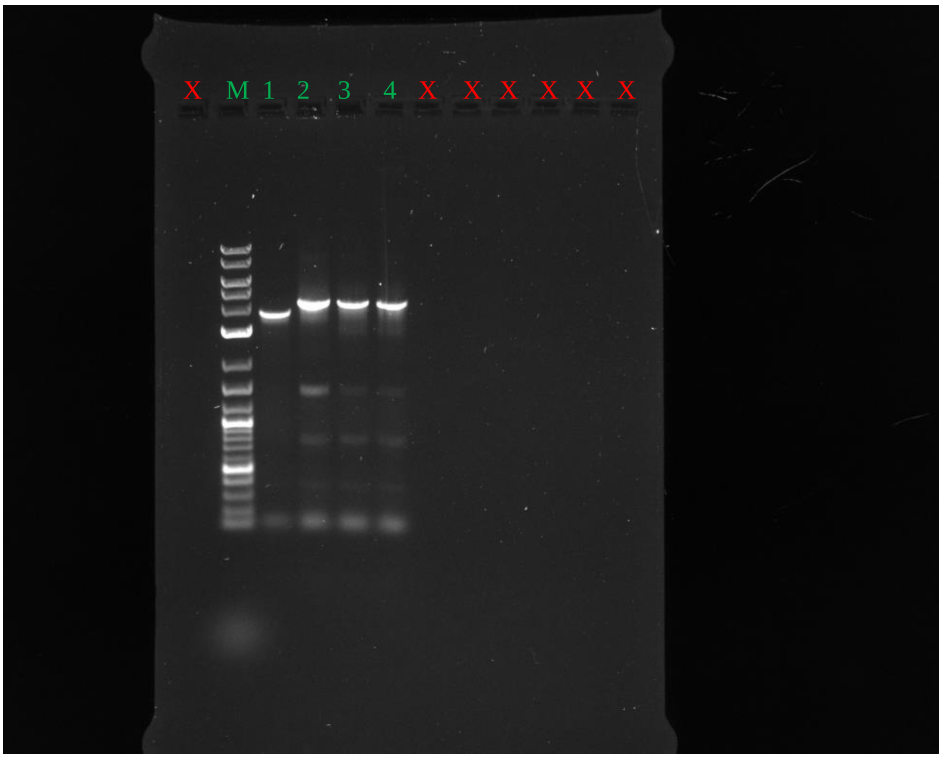

The above unedited gel image is present in Fig A1, Panel B. The image was taken with a Bio-Rad ChemiDoc Touch imagine system. Lanes with a red X are not used in the final Fig S1 image. Abbreviations: M-DNA marker (New England Biolabs, Catalog number: N3272S), 1- WT, 2 – Δ*fhpA* 1, 3 – Δ*fhpA* 4, 4 – Δ*fhpA* 5.

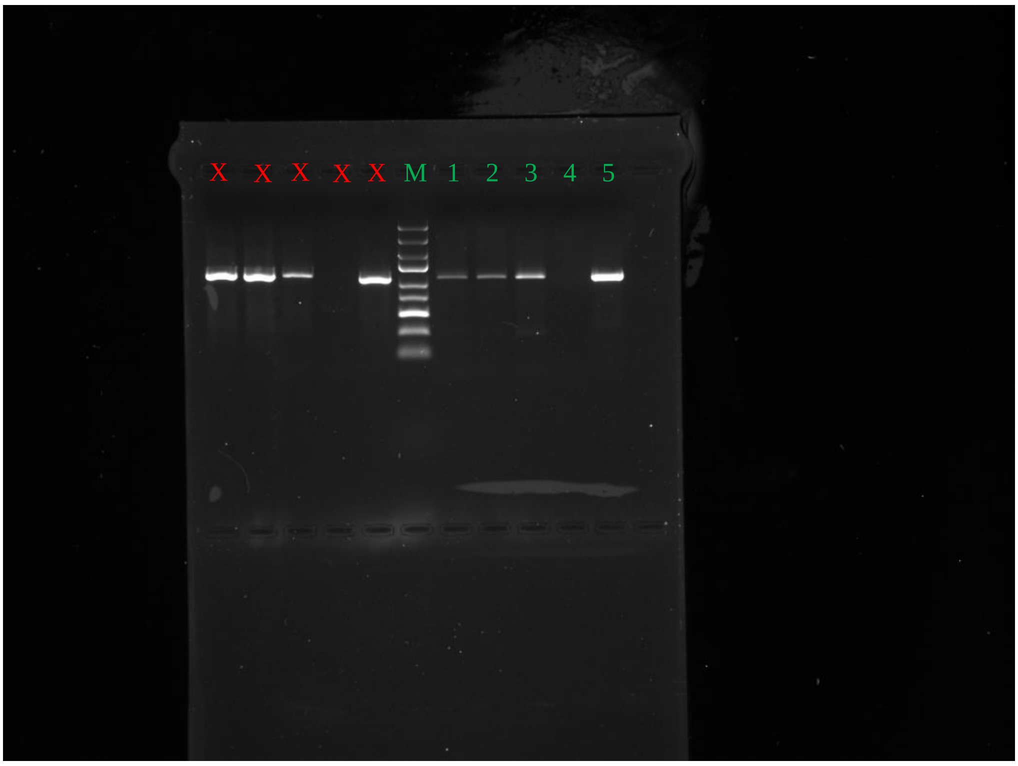

The above unedited gel image is present in Fig A1, Panel D. The image was taken with a Bio-Rad ChemiDoc Touch imagine system. Lanes with a red X are not used in the final Fig S1 image. Abbreviations: M-DNA marker (Thermo Scientific, Catalog number: SM1553), 1- OE*fhpA* B-1, 2 –OE*fhpA* 4, 3 – OE*fhpA* 8, 4 –WT, pOE fhpA
